# Cardiorenal Syndrome: Ebony and Ivory

**DOI:** 10.3390/diagnostics13091539

**Published:** 2023-04-25

**Authors:** Nans Florens

**Affiliations:** Service de Néphrologie, CHU de Strasbourg, Faculté de Médecine, Université de Strasbourg, Team 3072 “Mitochondria, Oxidative Stress and Muscle Protection”, Translational Medicine Federation of Strasbourg (FMTS), INI-CRCT (Cardiovascular and Renal Trialists), F-CRIN Network, F-67000 Strasbourg, France; nans.florens@chru-strasbourg.fr

The kidney and cardiovascular systems are closely interconnected and interact continuously in both physiological and pathological conditions. The dysfunction of one system can result in the dysfunction of the other, and this complex interplay has been named “cardiorenal syndrome”.

“Ebony and Ivory Live Together in Perfect Harmony Side by Side on my Piano Keyboard, oh Lord, Why don’t We?”

Cardiorenal syndrome, much as the classic duet “Ebony and Ivory” by Paul McCartney and Stevie Wonder [1], reminds us of the intricate interplay between two seemingly different entities and the harmony that can be achieved when they work together seamlessly. Just as the notes on a piano keyboard can harmonize to produce beautiful music, the interplay between the kidney and cardiovascular systems can result in a healthy physiological state. However, when this delicate balance is disrupted, it can lead to the cacophony that is cardiorenal syndrome—a condition that can produce a range of adverse outcomes from mild dysfunction to life-threatening complications.

Originally, cardiorenal syndrome referred to kidney failure leading to therapeutic failure in patients with severe heart insufficiency [2]. However, in 2010, the Acute Dialysis Quality Initiative redefined this syndrome to include five different types, depending on the initial organ that was injured [3]. The first two types (I and II) describe cardio-to-renal dysfunction, either acute or chronic, while the third and fourth types describe renal-to-cardiac dysfunction. The fifth type, induced by systemic conditions that can injure both organs in a similar manner, is characterized by simultaneous cardiorenal dysfunction. Although this classification is widely used in clinical practice, it has been criticized for its limitations, such as the difficulty in determining which organ was first affected in acute situations, its lack of correspondence with pathophysiological conditions, and its inability to account for acute-to-chronic lesions in both organs. As a result, in 2018, a new paradigm for cardiorenal pathology was proposed, introducing the concept of a pathophysiological continuum between the two organs with shared pathways of dysfunction that lead to increased fibrosis and dysfunction [4]. Thus, it is essential to redefine what is meant by cardiorenal syndrome, considering both the acute relationship between the two organs, which mainly involves hemodynamics and diuretic resistance, and the pathological crosstalk that leads to long-term dysfunction. There is a great deal of research that needs to be conducted to increase our understanding of cardiorenal pathology. For instance, questions remain about the primary diuretic resistance mechanisms in acute cardiac failure [5], whether an increased serum creatinine level is an appropriate marker for kidney dysfunction in acute cardiac conditions, and whether cardioprotective therapeutics posology should not be based solely on serum creatinine. It is important to note that a number of renal injury markers may be useful in diagnosing cardiorenal syndrome. More modern markers for estimating renal function, such as cystatin C, or tubular injury markers such as KIM-1 and NGAL, as well as albuminuria (a predominantly glomerular marker) and cell cycle arrest markers such as those found in NephroCheck (TIMP-2 and IGFBP7), could be employed. The main challenge lies in determining which part of the kidney is affected in cardiac situations, and, thus, which markers would be most appropriate. To date, none have truly demonstrated a utility that would warrant their widespread adoption in routine practice. In addition, new pathways of cardiorenal crosstalk have been identified in long-term cardiac damage following acute kidney injury, such as Galectin-3 [6], a monocyte marker, and the IL33/ST2 axis [7], which have been extensively studied in both organs independently. Furthermore, in chronic kidney disease, retinol, and retinol binding protein 4 accumulation has been linked to dysfunction of the internal clock of circulating monocytes that express G protein-coupled receptor 68 and are involved in cardiac remodeling via increased chemotaxis and inflammatory cytokine production in myocardial muscle [8]. These findings underscore the need for continued research into the pathophysiology of cardiorenal syndrome and the quest for novel cardiorenal risk markers extends to exploring unconventional, unusual, and innovative pathways.

Then, two main contingencies can be considered in the context of cardiorenal syndrome. The first pertains to the short-term adaptation and maladaptation of both organs in response to stress, with respect to factors such as hemodynamics and contractility for the heart, and hemodynamics and tubular function for the kidneys. The work of Dr. Testani and colleagues, for instance, has significantly contributed to our understanding of risk factors for diuretic resistance and the importance of focusing on the clinical relevance of our cardiac decongestion efforts rather than concentrating on creatinine levels. Indeed, an increase in creatinine is actually a favorable prognostic factor in heart failure when employing decongestion strategies [9], and elevated levels do not affect the long-term benefits of RAAS blockers on patient outcomes [10]. The second contingency concerns the long-term scars that may arise due to organ injury and/or dysfunction over time.

In conclusion, the complex interplay between the kidney and cardiovascular systems in cardiorenal syndrome opens up exciting new possibilities for understanding the pathophysiological pathways and developing potential treatments. With this Special Issue, we have aimed to redefine cardiorenal syndrome and explore its various aspects, including diuretic resistance, long-term fibrosis pathways, potential biomarkers for kidney dysfunction, and promising therapeutic approaches. The pursuit of improved prognostic and diagnostic markers in cardiorenal situations could potentially guide the more appropriate use of therapeutics such as SGLT2 inhibitors or antifibrotic agents (e.g., finerenone and RAAS blockers). These new insights and potential treatments hold promise for improving the lives of patients with cardiorenal syndrome.
